# LncRNA CRART16/miR-122-5p/FOS axis promotes angiogenesis of gastric cancer by upregulating VEGFD expression

**DOI:** 10.18632/aging.204078

**Published:** 2022-05-10

**Authors:** Junling Zhang, Xiaocong Pang, Lili Lei, Jixin Zhang, Xiaoqian Zhang, Ziyi Chen, Jing Zhu, Yong Jiang, Guowei Chen, Yingchao Wu, Tao Wu, Yisheng Pan, Yucun Liu, Yimin Cui, Xin Wang

**Affiliations:** 1 Department of General Surgery, Peking University First Hospital, Beijing 100034, China; 2 Institute of Clinical Pharmacology, Peking University, Beijing 100034, China; 3 Department of Pharmacy, Peking University First Hospital, Beijing 100034, China; 4 Department of Pathology, Peking University First Hospital, Beijing 100034, China; 5 Department of General Surgery, The Cancer Hospital of the Chinese Academy of Medical Sciences and China National Cancer Center, Beijing 100034, China; 6 Liver Transplantation Center, The First Affiliated Hospital of Nanjing Medical University, Nanjing 210029, China

**Keywords:** gastric cancer, angiogenesis, microRNA, long non-coding RNA, CRART16

## Abstract

Background: We previously identified a novel lncRNA, CRART16, that could induce cetuximab resistance in colorectal cancer cells. This study explored the relationship of CRART16 expression to gastric cancer progression and the molecular mechanisms involved.

Methods: We evaluated CRART16 expression in gastric cancer tissues and adjacent normal tissues from the TCGA database and our hospital. Besides, we assessed its relationship with the overall survival (OS) of patients with gastric cancer. The effects of CRART16 on gastric cancer angiogenesis were determined by endothelial tube formation assay, spheroid sprouting assay, HUVEC invasion assay, and chick embryo chorioallantoic membrane (CAM) assay. The involvement of the lncRNA CRART16/miR-122-5p/FOS axis was analyzed by western blotting and dual-luciferase reporter assay. The functions of CRART16 were confirmed in xenograft mouse models.

Results: We found that CRART16 was substantially overexpressed in gastric cancer tissues compared with normal tissues, based on the TCGA database and our clinical samples. High expression of CRART16 correlated with more advanced tumor stages and poor prognosis. Overexpression of CRART16 in gastric cancer cells promoted proliferation, colony formation, angiogenesis, and bevacizumab resistance *in vitro*, and it promoted tumor growth and angiogenesis *in vivo*, and vice versa. CRART16 was found to downregulate miR-122-5p by acting as a sponge, upregulating the target oncogene FOS. Afterward, the increased FOS expression led to the upregulation of VEGFD.

Conclusion: Our findings demonstrate that CRART16 promotes angiogenesis *in vitro* and *in vivo*, and CRART16 is a prognostic marker and therapeutic target in gastric cancer.

## INTRODUCTION

Gastric cancer is the fifth most prevalent malignancy and the fourth most common cause of cancer death worldwide. In 2020, there were over one million newly diagnosed patients with gastric cancer, with an estimated 769,000 deaths globally [1]. Nearly two-thirds of the global incident cases occur in developing countries [2].

Chemotherapy combined with targeted therapy can prolong the survival of patients with multiple advanced cancers. Since tumor vascularization is closely related to tumor growth, drug resistance, and metastatic spread of cancers, targeted anti-angiogenic therapies are among the most promising treatment modalities. Therefore, many drug developers have begun to focus on signaling molecules that modulate angiogenesis. Despite these advances, the median overall survival (OS) remains shorter than 12 months for patients with metastatic or unresectable gastric cancer [3]. The main challenge of anti-angiogenic targeted therapy is drug resistance.

One of the causes of such resistance may be long non-coding RNAs (lncRNAs) [4], which do not encode any protein but play regulatory functions by serving as signals, guides, decoys, or scaffolds [5]. The dysregulation of specific lncRNAs is closely involved in the development of various cancers, and such alterations can be easily detected in body fluids. The characteristics of lncRNAs demonstrate that lncRNAs may serve as non-invasive diagnostic biomarkers and prognostic biomarkers [6, 7]. LncRNA CRART16 (ENST00000564193.1) is 744 bp, and it contains two introns and three exons and lies on chromosome 16. Our previous study showed that CRART16 was upregulated significantly in a cetuximab-resistant colon cancer cell line [8]. Additionally, lncRNA CRART16 overexpression repressed cetuximab-induced apoptosis in colon cancer cells by enhancing the expression of HER3 [8]. Therefore, we wondered whether CRART16 contributes to gastric cancer or influences prognosis.

The present study explored potential correlations between CRART16 expression and clinicopathologic characteristics of patients with gastric cancer. Furthermore, we used *in vitro* and *in vivo* models to examine whether CRART16 influences tumor angiogenesis during the disease.

## RESULTS

### Upregulated CRART16 expression correlated with adverse clinicopathologic features and lower OS in gastric cancer patients

**Figure 1:**
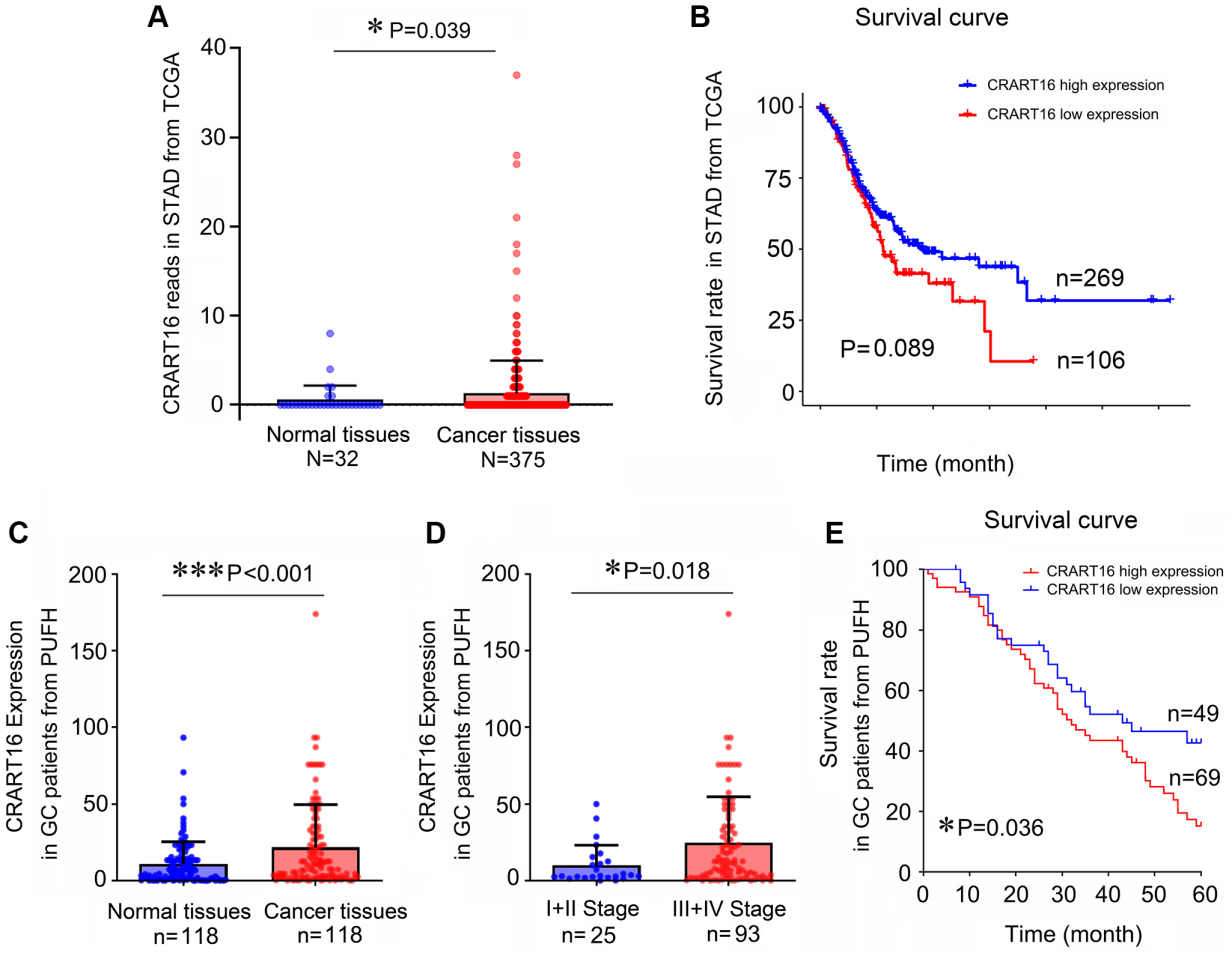
**Gastric cancer tissues express a higher level of CRART16, and the higher level of CRART16 correlates with lower overall survival of gastric cancer patients.** (**A**) CRART16 expression levels in normal and cancerous tissues from the TCGA-STAD dataset (^*^P = 0.039). (**B**) Survival curves of patients with gastric cancer from TCGA-STAD dataset based on their CRART16 expression level (*P* = 0.089). (**C**) Compared with normal gastric tissues, cancerous tissues from our institution expressed a higher level of CRART16 (^***^P < 0.001). (**D**) Cancerous tissues from stage III/IV gastric cancer expressed significantly higher levels of CRART16 than those from stage I/II gastric cancer (^*^P = 0.018). (**E**) The survival curves of gastric cancer patients from our institution were based on their CRART16 expression (log-rank test: χ^2^ = 4.394, ^*^P = 0.036).

In this study, TCGA (The Cancer Genome Atlas) data mining showed that gastric cancer tissues expressed a significantly higher level of CRART16 than normal tissues (*P* = 0.039; Figure 1A). Additionally, survival analysis indicated that patients with lower CRART16 had better OS rates than patients with higher CRART16 expression, but not significantly (*P* = 0.089; Figure 1B). Next, we aimed to confirm these observations using samples of gastric cancer patients from our institution. The result of quantitative real-time PCR (qRT-PCR) demonstrated that the relative level of CRART16 in gastric cancer tissues was remarkably higher than that in normal tissues (^***^P < 0.001; Figure 1C). Furthermore, malignant tissues from stage III/IV gastric cancer expressed significantly higher levels of CRART16 than those from stage I/II gastric cancer (^*^P = 0.018; Figure 1D). Gastric cancer patients were categorized into low or high CRART16 expression groups based on whether the level in gastric cancer tissues was less than or at least as high as the mean level of normal tissues, respectively. High CRART16 expression in cancerous tissue was associated with significantly lower OS (^*^P = 0.036; Figure 1E).

### Gastric cancer cell lines express higher levels of CRART16

**Figure 2:**
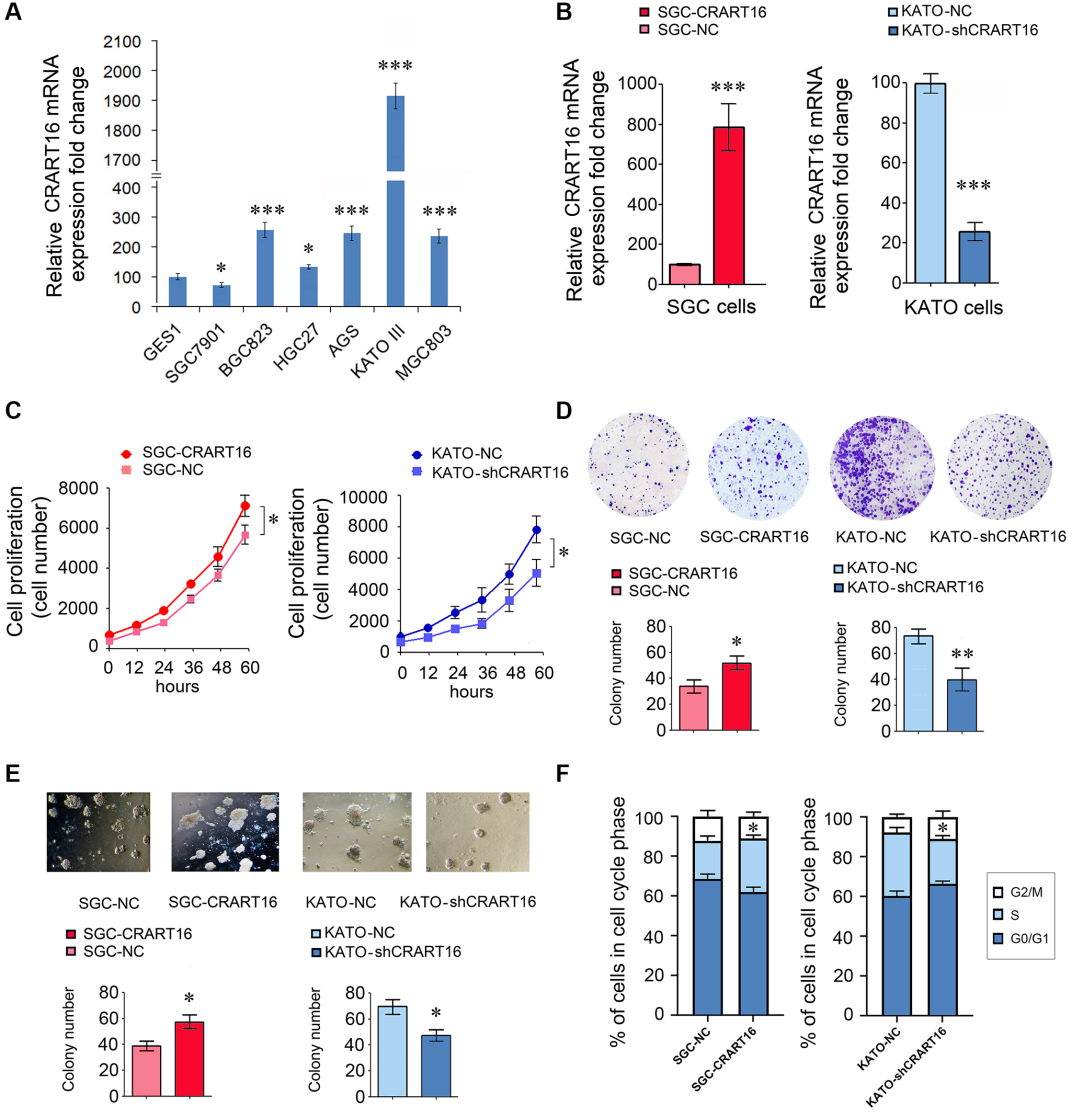
**CRART16 promotes the growth rate, clonogenicity, and the cell cycle progression of gastric cancer cells.** (**A**) CRART16 expressions were examined in six kinds of gastric cancer cell lines and one normal gastric cell line using qRT-PCR (^*^P < 0.05, ^***^P < 0.001). (**B**) The expression of CRART16 was examined by qRT-PCR in SGC-7901 cells transfected with CRART16 or negative control lentiviruses. CRART16 expression was also examined in KATO-III cells transfected with CRART16 shRNA or negative control plasmids using qRT-PCR. GAPDH served as a loading control (^***^P < 0.001). (**C**) The CCK-8 assay determined the growth rate of gastric cancer cells (^*^P < 0.05). (**D**) Representative images of cell colonies formed by the indicated gastric cancer cells on the 14^th^ day after seeding (^*^P < 0.05). (**E**) Representative images of cell colonies formed in Matrigel by the indicated gastric cancer cells on the 14^th^ day after seeding (^*^P < 0.05). (**F**) Cell cycle distribution and analysis of S phase of indicated gastric cancer cells (^*^P < 0.05). All data are presented as mean ± SD from three separate experiments.

Compared with normal gastric epithelial cell line GES-1, five gastric cancer cell lines expressed relatively higher levels of CRART16 (Figure 2A). Among the cancer lines, the highest CRART16 expression level was detected in KATO-III cells and the lowest in SGC-7901 cells (^*^P < 0.05, ^***^P < 0.001; Figure 2A). Therefore, the SGC-7901 cell line and KATO-III cell line were selected for experiments in the next step.

### CRART16 overexpression promotes the proliferation, clonogenicity, and bevacizumab resistance of gastric cancer cells

Our results showed that, compared with negative control (NC) cells, SGC-CRART16 expressed a higher level of CRART16 (^***^P < 0.001; Figure 2B). Moreover, the overexpression of CRART16 increased the proliferation significantly (^*^P < 0.05; Figure 2C). Besides, the overexpression of CRART16 promoted the clonogenicity of gastric cancer cells significantly (^*^P < 0.05; Figure 2D, 2E).

Conversely, the relative expression of CRART16 was lower in KATO-shCRART16 cells than that in KATO-NC cells (^***^P < 0.001; Figure 2B). Furthermore, downregulation of CRART16 decreased the cellular growth rate (^*^P < 0.05; Figure 2C) and clonogenicity significantly (^*^P < 0.05, ^**^P < 0.01; Figure 2D, 2E).

Next, we performed a cell cycle analysis using flow cytometry to determine whether CRART16 expression promotes cell growth rate. Compared with negative control cells, the percentage of SGC-CRART16 cells in the S-phase was increased significantly (^*^P < 0.05; Figure 2F). Conversely, knocking down CRART16 in KATO-shCRART16 cells depressed the percentage of cells in the S-phase (^*^P < 0.05; Figure 2F).

### CRART16 overexpression suppresses bevacizumab-induced apoptosis of gastric cancer cells

**Figure 3:**
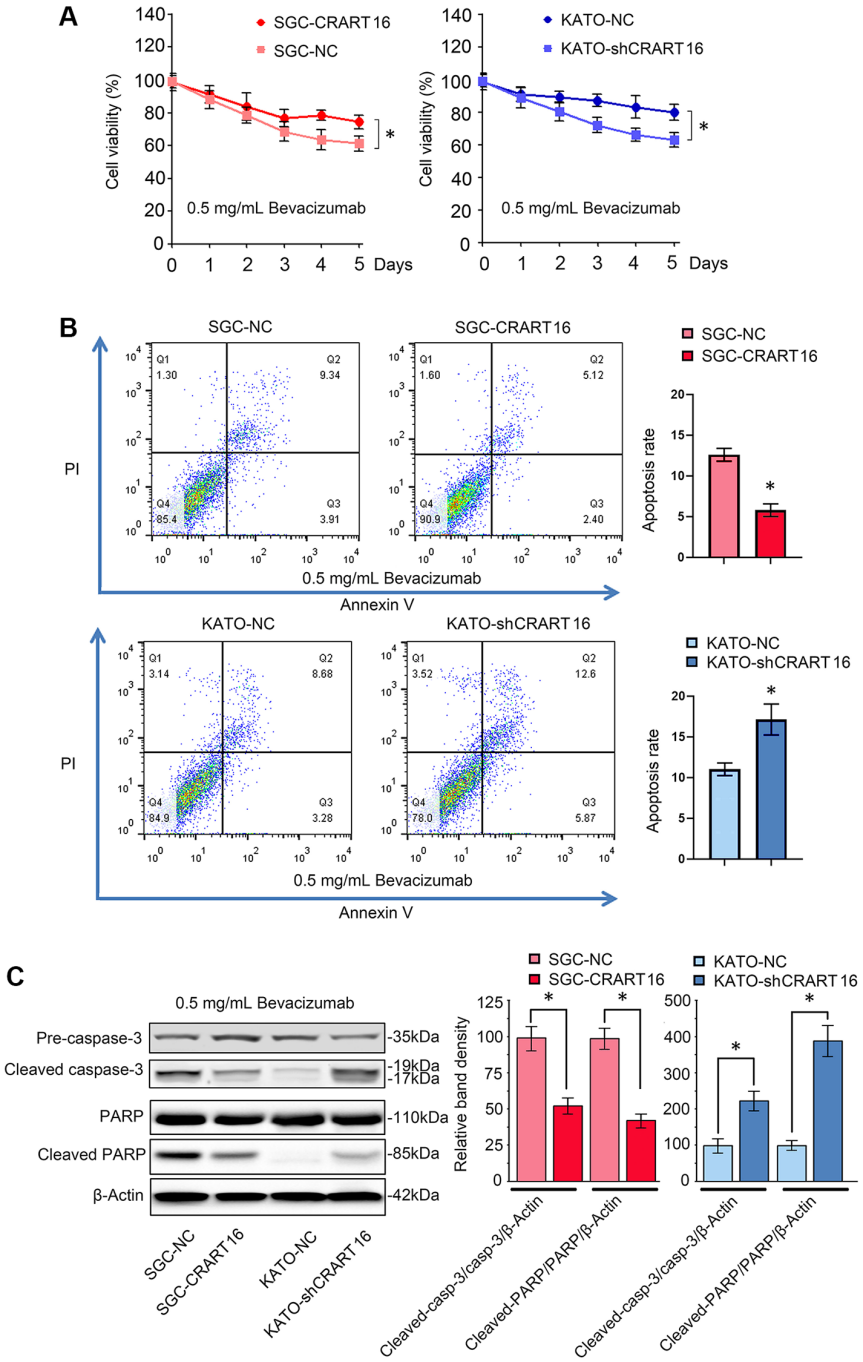
**CRART16 overexpression reverses bevacizumab-induced apoptosis.** (**A**) The CCK8 assay was carried out to evaluate the viability of different gastric cancer cells treated with bevacizumab for five days (^*^P < 0.05). (**B**) Flow cytometry showing the apoptosis rate of indicated gastric cancer cells treated with bevacizumab for 48 hours (^*^P < 0.05). (**C**) Based on western blotting, the relative expression levels of cleaved caspase-3 and cleaved PARP in indicated gastric cancer cells treated with bevacizumab for 48 hours. β-Actin was used as a loading control (^*^P < 0.05). All data are presented as mean ± SD from three separate experiments.

Overexpression of CRART16 increased the viability of gastric cancer cells after 5-day treatment with bevacizumab (0.5 mg/mL), suggesting that CRART16 promoted bevacizumab resistance (^*^P < 0.05; Figure 3A). Additionally, the result of flow cytometry indicated that bevacizumab led to significantly lower apoptosis rates in SGC-CRART16 cells than in SGC-NC cells. Conversely, downregulation of CRART16 in gastric cancer cells effectively improved apoptosis rates induced by bevacizumab (^*^P < 0.05; Figure 3B). Western blotting assays proved that CRART16-overexpressing depressed the levels of cleaved caspase-3 and cleaved PARP induced by bevacizumab. Conversely, CRART16-silencing yielded opposite results (^*^P < 0.05; Figure 3C).

### CRART16 overexpression promotes angiogenesis induced by gastric cancer

**Figure 4:**
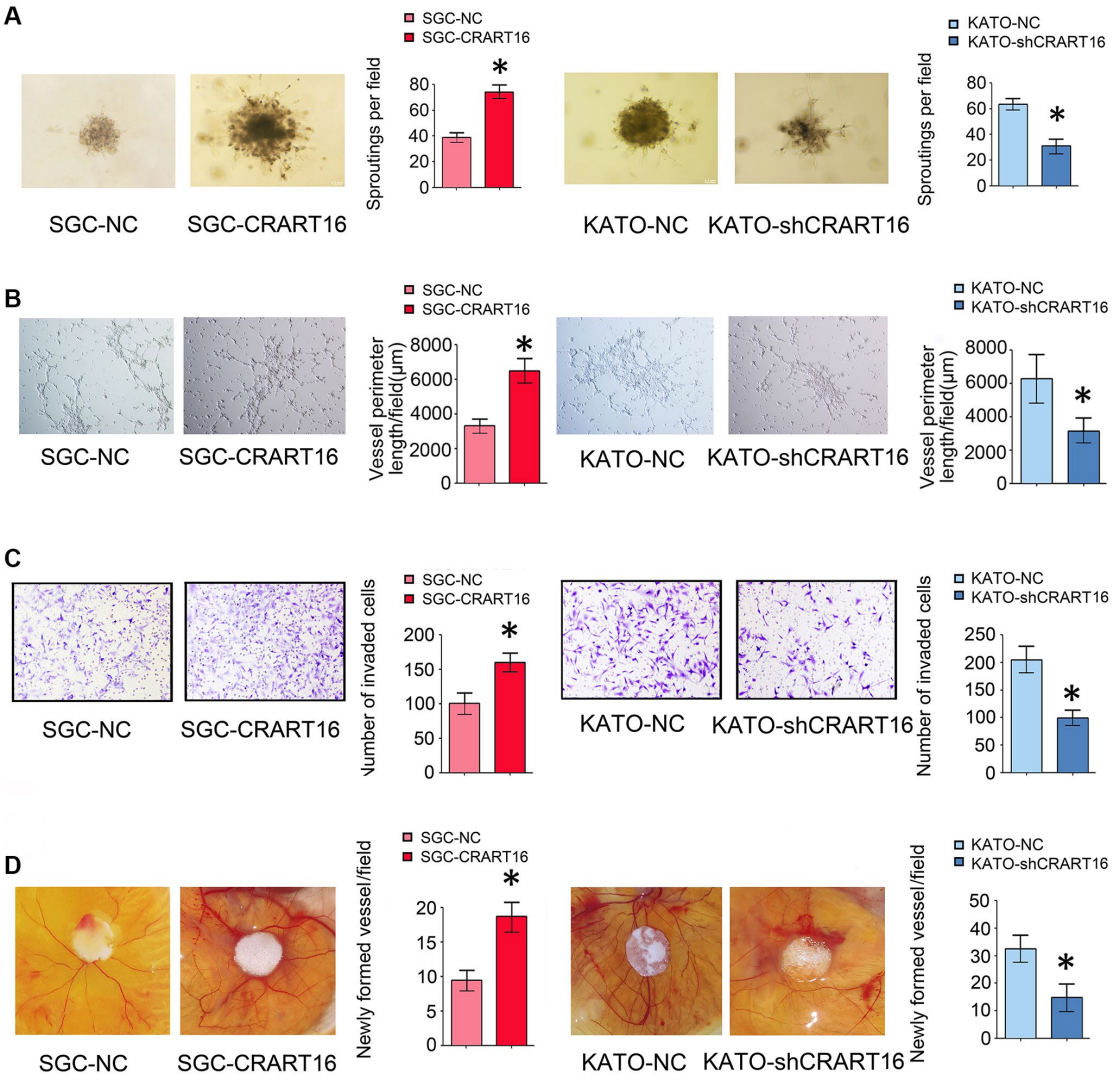
**Overexpression of CRART16 in gastric cancer cells promotes angiogenesis.** (**A**) 3D spheroid sprouting assay showed that conditioned medium from CRART16-overexpressing gastric cancer cells induced more prominent spheroid sprouting of HUVECs, and vice versa (^*^P < 0.05). (**B**) Endothelial cell tube formation assay showed that conditioned medium from CRART16-overexpressing gastric cancer cells induced a more significant tube-like structures formation of HUVECs. Conversely, CRART16-silencing had the opposite effects on tube-like formation (^*^P < 0.05). (**C**) The invasive capability of HUVECs was determined using the transwell invasion assay (^*^P < 0.05). (**D**) Chick chorioallantoic membrane (CAM) assays. Newly formed vessels per field were quantified (^*^P < 0.05). All data are presented as mean ± SD from three separate experiments.

The effects of CRART16 on the angiogenic ability of human umbilical vein endothelial cells (HUVECs) were evaluated using spheroid sprouting, tube formation, and invasion assays. HUVECs were pretreated for 36 hours with a conditioned medium from CRART16-overexpressing gastric cancer cells, CRART16-silenced gastric cancer cells, or their corresponding negative control cells. Treatment with CRART16-overexpressing conditioned medium induced a more significant tube-like structures formation of HUVECs and more prominent spheroid sprouting (^*^P < 0.05, Figure 4A, 4B). It also enhanced the invasive capacity of HUVECs in the transwell assay (^*^P < 0.05, Figure 4C). Meanwhile, CRART16-silenced gastric cancer cells’ conditioned medium exerted the opposite effects on HUVECs (^*^P < 0.05, Figure 4C). Similarly, CRART16-overexpressing gastric cancer cells led to more significant microvessel formation in the chick chorioallantoic membranes (CAM) assay than negative control cells. Conversely, CRART16-silenced gastric cancer cells had the opposite effects on microvessels formation in the CAM assay (^*^P < 0.05; Figure 4D).

### CRART16 downregulates miR-122-5p and upregulates FOS, VEGFD

**Figure 5:**
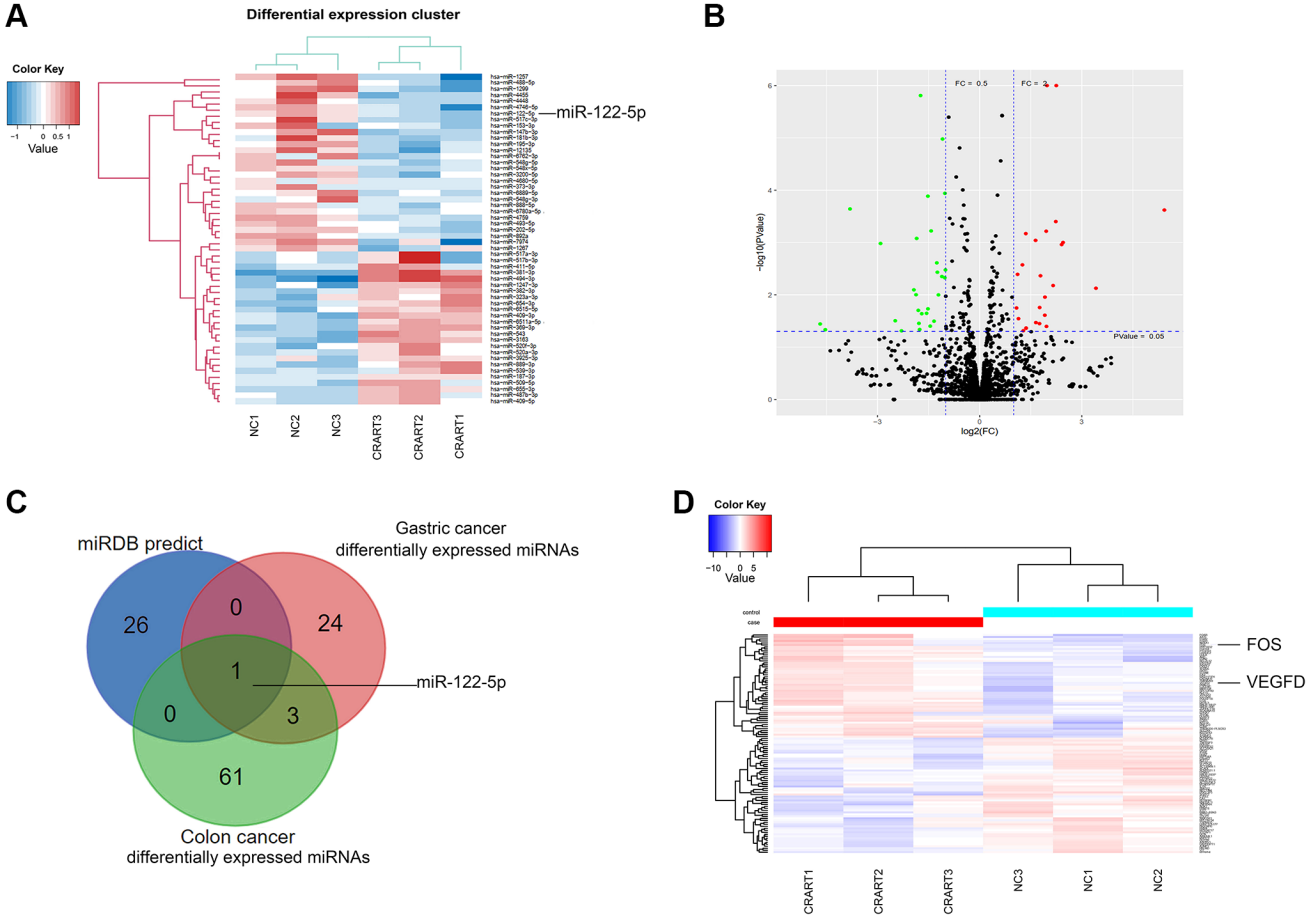
**RNA profiling of CRART16-overexpressing gastric cancer.** (**A**) Heatmaps of microRNAs (miRNAs) showing the top differentially expressed genes between SGC-CRART16 and SGC-NC cells (*n* = 3). (**B**) A volcano plot showing the differentially expressed individual miRNAs in SGC-CRART16 cells. The miRNAs significantly upregulated in the SGC-CRART16 group are shown in red; the miRNAs significantly downregulated are shown in green. (**C**) Venn diagram showing the intersection of differentially expressed miRNAs from gastric cancer and colon cancer cell lines with the potential miRNA targets from miRwalk prediction. The most probable target of CRART16 was identified to be miR-122-5p. (**D**) Heatmaps of mRNAs showing the top differentially expressed genes between SGC-CRART16 and SGC-NC cells (*n* = 3).

Next-generation sequencing showed that, compared with negative control cells, 24 microRNAs (miRNAs) were downregulated, and 25 miRNAs were upregulated in CRART16-overexpressing gastric cancer cells (Figure 5A, 5B). We combined the potential miRNA targets from miRwalk prediction and the differentially expressed miRNAs sets from CRART16-overexpressing gastric cancer cells and colon cancer cells using the Venn diagram. Venn analysis identified miR-122-5p as the most probable target of CRART16 (Figure 5C). Besides, the sequencing data showed that FOS and VEGFD expression were upregulated in CRART16-overexpressing gastric cancer cells (Figure 5D).

**Figure 6:**
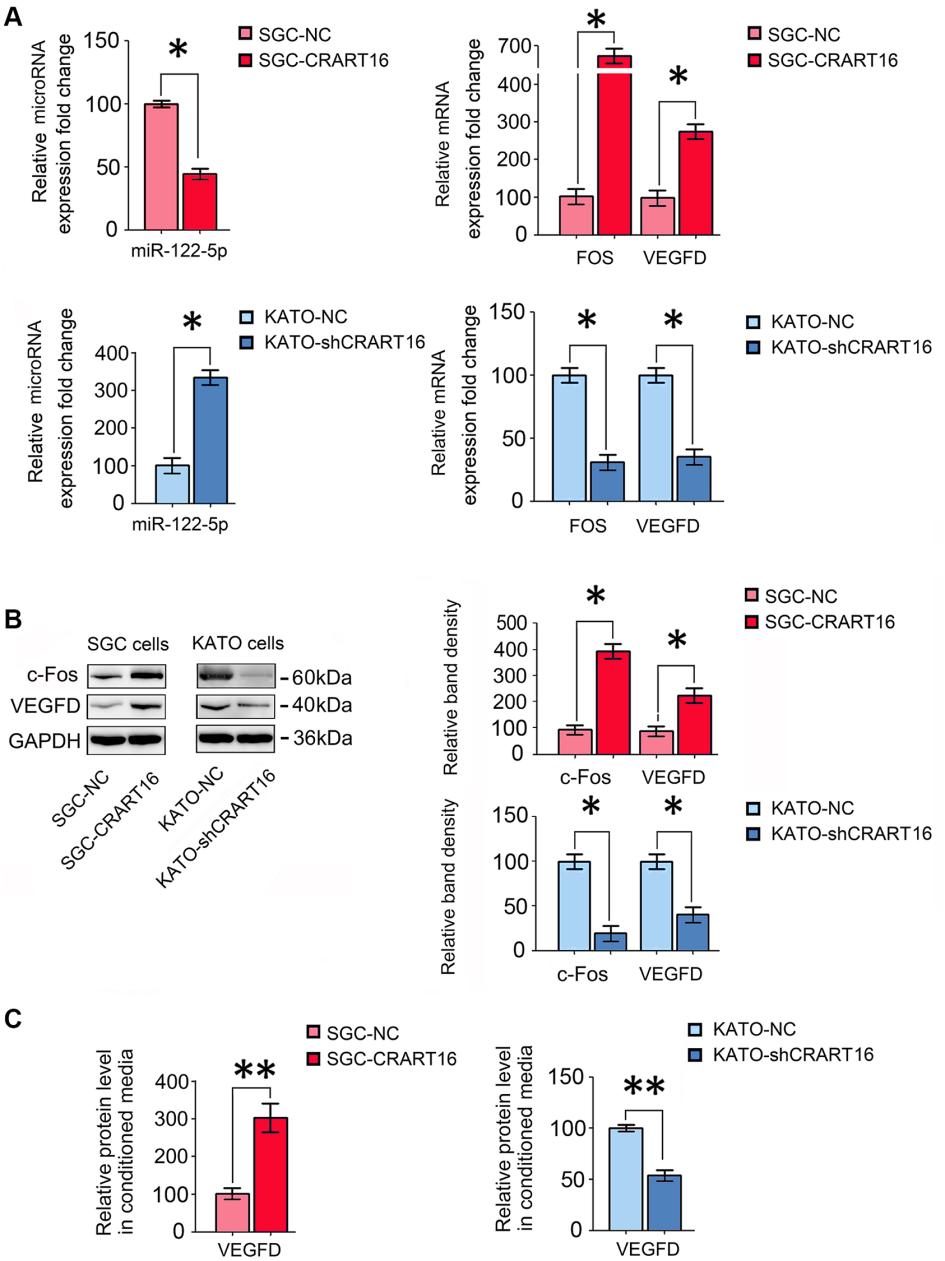
**CRART16 overexpression increases c-Fos and VEGFD expression by targeting miR-122-5p.** (**A**) The relative levels of miR-122-5p and mRNAs encoding FOS and VEGFD in different gastric cancer cell lines were analyzed by qRT-PCR (^*^P < 0.05). (**B**) The relative levels of c-Fos and VEGFD in different gastric cancer cell lines were determined by western blotting. GAPDH was used as a loading control (^*^P < 0.05). (**C**) The expression levels of VEGFD in conditioned media from different types of gastric cancer cells were determined by ELISA (^**^P < 0.01). All data are presented as mean ± SD from three separate experiments.

The sequencing results were confirmed by qRT-PCR and western blotting assays. Compared with the negative control cells, CRART16-overexpressing gastric cancer cells expressed a lower level of miR-122-5p and higher FOS and VEGFD (^*^P < 0.05; Figure 6A, 6B). Compared with negative control cells, ELISA results demonstrated that VEGFD expression was upregulated in conditioned media from CRART16- overexpressing gastric cancer cells (^**^P < 0.01; Figure 6C). Conversely, CRART16-silencing had the opposite effects on the expression levels of miR-122-5p, FOS, and VEGFD (^*^P < 0.05, ^**^P < 0.01; Figure 6A–6C).

### CRART16 promotes angiogenesis and bevacizumab resistance through the miR-122-5p/c-FOS axis

**Figure 7:**
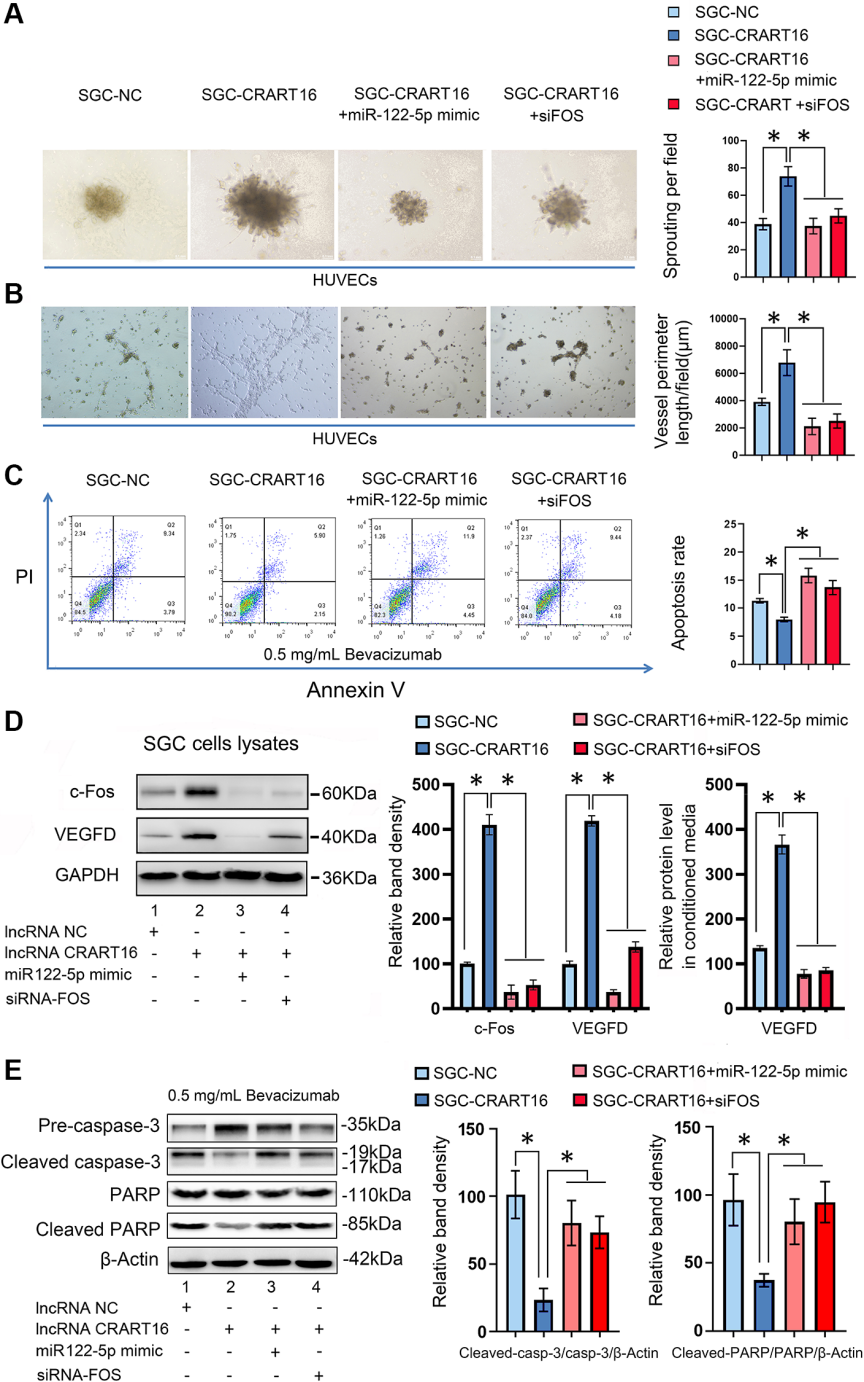
**CRART16 promotes angiogenesis and bevacizumab resistance by regulating the miR-122-5p/c-Fos axis.** (**A**) Effects of conditioned medium on spheroid sprouting of HUVECs (^*^P < 0.05). (**B**) Effects of indicated conditioned medium on capillary formation by HUVECs (^*^P < 0.05). (**C**) The apoptosis rate of indicated cells treated with bevacizumab for 48 hours (^*^P < 0.01). (**D**) Levels of c-Fos and VEGFD were determined by western blotting. GAPDH served as a loading control (^*^P < 0.05). The expression levels of VEGFD in conditioned media from different kinds of gastric cancer cells were determined by ELISA (^*^P < 0.05). (**E**) Based on western blotting, the relative expression levels of cleaved caspase-3 and cleaved PARP in indicated gastric cancer cells treated with bevacizumab for 48 hours. β-Actin served as a loading control (^*^P < 0.05). All data are presented as mean ± SD from three separate experiments.

To characterize the effects of miR-122-5p and FOS on CRART16-mediated angiogenesis and bevacizumab resistance, the miR-122-5p mimics or siRNAs targeting FOS were separately transfected into SGC-CRART16 cells (Figure 7). HUVECs cultured in a conditioned medium from SGC-CRART16 cells showed more significant tube-like structures and spheroid sprouts than HUVECs cultured in a conditioned medium from the SGC-CRART16 cells transfected with the miR-122-5p mimics or FOS siRNAs (^*^P < 0.05; Figure 7A, 7B). In addition, the miR-122-5p mimics or FOS siRNAs reversed bevacizumab resistance induced by CRART16 overexpression in gastric cancer cells (^*^P < 0.05; Figure 7C). Based on western blotting and ELISA assays, they also reduced c-Fos and VEGFD protein levels (^*^P < 0.05; Figure 7D). Western blotting assays subsequently confirmed the apoptosis results from flow cytometry assays above. Western blotting results demonstrated that CRART16 overexpression depressed the expression of cleaved PARP and cleaved caspase-3 induced by bevacizumab. Meanwhile, miR-122-5p mimics transfection or FOS siRNAs transfection reversed these results (^*^P < 0.05; Figure 7E).

### CRART16 functions as a miR-122-5p sponge

**Figure 8:**
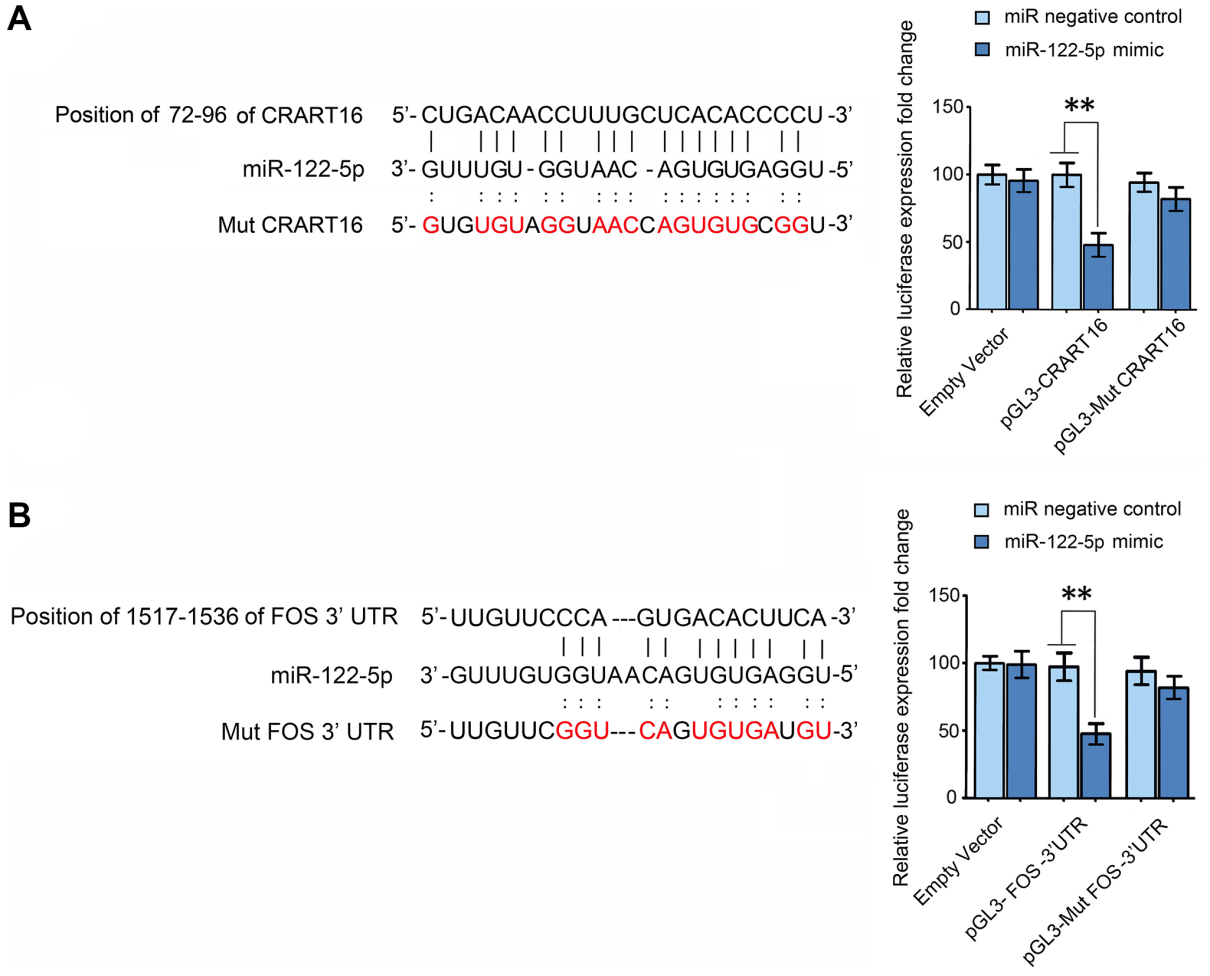
**CRART16 functions as a miR-122-5p sponge, and miR-122-5p targets FOS mRNA.** (**A**) The predicted binding site between CRART16 and miR-122-5p is illustrated. The luciferase activities were determined after co-transfection with either miR-122-5p mimics or negative control mimics and pGL3 encoding wild-type or mutated (Mut) CRART16 (^**^P < 0.01). (**B**) The putative binding site between miR-122-5p and the 3’-UTR of FOS mRNA is illustrated. The luciferase activities were determined after co-transfection with miR-122-5p mimics or negative control mimics and pGL3-FOS carrying the wild-type or mutated (Mut) 3’UTR (^**^P < 0.01). All data are presented as mean ± SD from three separate experiments.

RNA22 v2 microRNA target detection and miRanda were used to spot potential base-pairing regions between CRART16 and potential target microRNAs. CRART16 was predicted to harbor one binding site for miR-122-5p (Figure 8A). The interaction between miR-122-5p and CRART16 was tested by dual-luciferase reporter assays (Figure 8A). The HEK293T cells were co-transfected with luciferase reporter plasmids and miR-122-5p mimics or negative control. The luciferase activities of reporter plasmids with wild-type CRART16 were remarkably depressed by the miR-122-5p mimics. However, the miR-122-5p mimics did not affect the luciferase activities of mutant reporters (^**^P < 0.01; Figure 8A). The results prove that CRART16 functions as a miR-122-5p sponge, which inhibits the activity of the miR-122-5p.

### MiR-122-5p targets FOS

Target-Scan predicted the 3′ untranslated region (UTR) of FOS mRNA to contain a binding site for miR-122-5p (Figure 8B). To validate whether miR-122-5p directly targets FOS, FOS 3′-UTRs with mutant or wild-type miR-122-5p binding sequences were cloned into the downstream of the luciferase reporter gene in pGL3 vectors (Figure 8B). The HEK293T cells were co-transfected with the reporter plasmids, and miR-122-5p mimics or negative control, the luciferase activities of reporters whose transcripts carried the wild type FOS 3′-UTR were significantly depressed by miR-122-5p mimics, but the luciferase activities of reporters whose transcripts carried the mutant 3′-UTR were unaffected (^**^P < 0.01; Figure 8B). The results above show that miR-122-5p depresses the expression of FOS by binding to the 3′-UTR of its mRNA.

### CRART16 promotes tumor growth and neovascularization in nude mouse models

**Figure 9:**
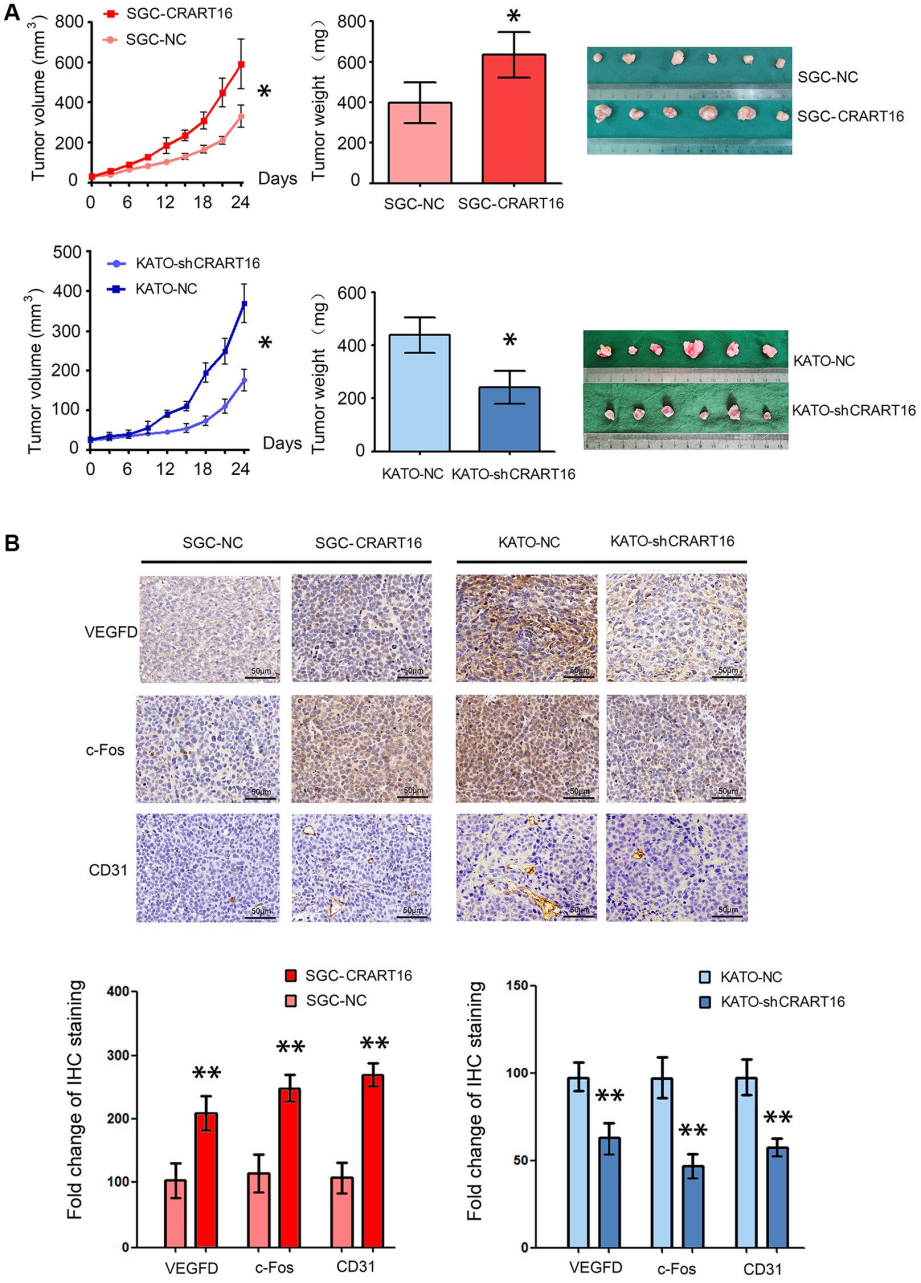
**CRART16 overexpression in gastric cancer cells accelerates tumor growth and upregulates angiogenesis in nude mouse models.** (**A**) Tumor volumes and tumor weights are illustrated as mean ± SD for six mice from different groups (^*^P < 0.05). (**B**) Representative micrographs and quantitation of immunostaining against c-Fos, VEGFD, and CD31 in different tumor sections. All data are presented as mean ± SD of quintuplicate determinations of six mice from different groups (^**^P < 0.01).

To confirm the function of CRART16 in nude mouse models, different types of gastric cancer cells were injected into the flanks of mouse models subcutaneously. As illustrated in Figure 9A, tumor growth, and tumor weights were significantly higher in the CRART16-overexpressing group than those in the negative control group (^*^P < 0.05; Figure 9A). In addition, the immunohistochemistry staining showed that, compared with negative control cells, the CRART16 overexpression group expressed higher levels of c-Fos, VEGFD, and CD31 significantly (^**^P < 0.01; Figure 9B). Conversely, CRART16-shRNA had the opposite effects on tumor growth, tumor weights, angiogenesis, and expressions of c-Fos, VEGFD, and CD31. These findings indicate that CRART16 overexpression promotes gastric tumor growth and tumoral angiogenic activity by upregulating c-Fos and VEGFD expression.

## DISCUSSION

With the advancement of high-throughput sequencing technologies, lncRNAs have been identified as essential factors regulating carcinogenesis. This study compared the gene expressions of cancerous tissues and adjacent normal tissues using sequencing data from TCGA and gastric cancer patients at our medical institution. The results showed that, compared with normal tissues, CRART16 was upregulated in cancerous tissues significantly. Additionally, increased expression of CRART16 was significantly associated with a poorer prognosis in patients with gastric cancer. Meanwhile, compared with normal gastric mucosa cells, five types of gastric cancer cells expressed a higher level of CRART16. Therefore, we hypothesized that CRART16 was a critical oncogene in gastric cancer.

Our further research found that overexpression of CRART16 promoted proliferation, clonogenicity, and cell cycle progression in gastric cancer cells. Additionally, CRART16 upregulation promoted bevacizumab resistance and decreased the apoptosis rate induced by bevacizumab. Furthermore, CRART16 promoted angiogenesis both *in vitro* and *in vivo*, and a conditioned medium from CRART16-overexpressing gastric cancer cells stimulated invasion of HUVECs.

Through bioinformatics prediction and validation in a dual-luciferase reporter assay, we found that CRART16 overexpression sponged miR-122-5p. Our results were consistent with several studies suggesting that miR-122-5p can protect against gastric cancer. For example, a study showed that gastric cancer tissues expressed a lower level of miR-122-5p than adjacent normal tissues [9]. At the same time, the gastric cancer cell lines expressed a lower level of miR-122-5p than that in the normal gastric epithelial cell line significantly [9]. Moreover, levels of miR-122 in gastric cancer patients were lower when distant metastases were present [10]. Additionally, higher levels of miR-122 in plasma correlated with a more favorable prognosis for gastric cancer [10]. Besides, miR-122-5p in exosome blocked gastric cancer cell proliferation, adhesion, invasion, and tumor growth [11].

By sponging miR-122-5p, we found that CRART16 upregulated FOS, which increased the expression of VEGFD, given that FOS increased the activity of the promoters of the VEGFD [12–14]. FOS is a proto-oncogene with an essential role in many kinds of cellular functions and is overexpressed in various cancers [15]. C-Fos is encoded by the FOS gene, and it is a crucial member of the activator protein 1 (AP-1) transcription factor. C-Fos has a variety of functions in regulating cell growth, differentiation, and metastasis [16]. In colon cancer cells, silencing the expression of c-Fos reduced the invasive capability of cancer cells and the number of lung metastases in mouse models [17]. A recent study proved that c-Fos played a critical role in metastasis in gastric cancer, and c-Fos binding sites of the MMP-9 promoter were activated by p38 [18]. A previous study had proved that c-Fos upregulated VEGFD directly [13]. VEGFD, also known as c-fos-induced growth factor (FIGF), is the newest member of the VEGF family [19]. VEGFD can bind to the receptor VEGFR-2 on vascular endothelial cells, and the activation of VEGFR-2 promotes angiogenesis, tumor growth, and metastasis [20]. Several studies have proved that VEGFD contributes to the angiogenesis of gastric cancer [20], and VEGFD is a biomarker of disseminated disease in gastric cancer patients [21].

Bevacizumab is a humanized monoclonal antibody that neutralizes all the human VEGF isoforms, and bevacizumab depresses VEGF-induced growth of endothelial cells [22]. Animal models showed that bevacizumab decreased the growth of xenograft tumors by depressing tumor neovascularization [23, 24]. Furthermore, a clinical trial has shown that bevacizumab has anti-malignancy activity in gastric cancer [25]. However, the overall survival time of patients with advanced gastric cancer is much shorter than expected due to the development of acquired drug resistance. VEGFD may be one of the mechanisms of drug resistance [26]. The present research demonstrates that the CRART16/miR-122-5p/FOS axis increases angiogenesis and promotes bevacizumab resistance by elevating VEGFD expression.

We used nude mouse xenograft models to confirm a role for the CRART16/miR-122-5p/FOS axis in regulating cancer cell growth and angiogenesis. CRART16 overexpression increased the size of xenograft tumors and the density of microvessels. It also increased the expression of c-Fos, VEGFD, and CD31. These findings support the idea that CRART16 promotes gastric cancer by increasing angiogenesis.

Our study presents several limitations. First, our clinical data showed that higher CRART16 expression levels in gastric cancer tissues were associated with poorer overall survival in gastric cancer patients. However, the data from the TCGA dataset showed that patients with lower CRART16 expression had better overall survival than patients with higher CRART16 expression, but not significantly. We suppose that the depth deficiency in transcriptional sequencing is one of the fundamental reasons for this inconsistency. Second, we did not get gastric cancer tissues from patients with bevacizumab resistance to further test the expression levels of CRART16 in drug-resistant tissues.

## CONCLUSIONS

**Figure 10:**
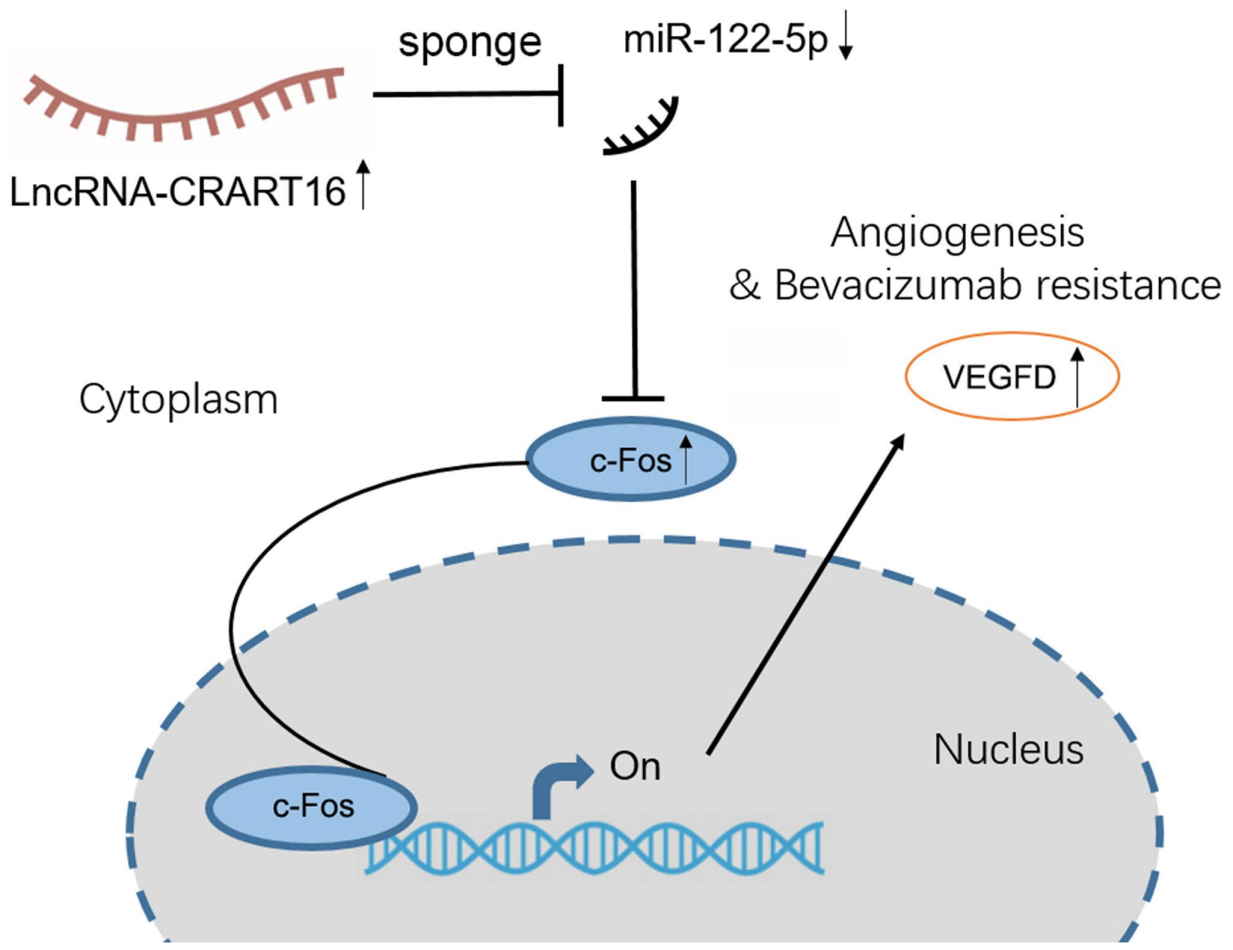
Proposed mechanism for how the axis involving lncRNA CRART16, miR-122-5p, and c-Fos drives angiogenesis and bevacizumab resistance in gastric cancer.

Our study demonstrates that CRART16 promotes angiogenesis and tumor proliferation in gastric cancer by acting as a miR-122-5p sponge and thereby upregulating c-Fos and VEGFD expression (Figure 10). Therefore, we suppose that CRART16 may be a valuable biomarker for predicting the prognosis of patients with gastric cancer and for developing anti-angiogenic drugs against the disease.

## MATERIALS AND METHODS

### Collection and processing of publicly available data

CRART16 expression data in gastric cancer tissues and clinical data of patients were analyzed from The Cancer Genome Atlas (TCGA) database. The data from 375 gastric cancer tissues and 32 normal tissues were used. The expression of CRART16 was analyzed with R version 4.0 [27]. In addition, OS was assessed using Kaplan-Meier curves and visualized using the “survival” package in R [28].

### Collection of tissues and data from patients at our hospital

This study analyzed samples that had been prospectively collected from 118 patients who had been diagnosed with pathologically diagnosed gastric cancer at Peking University First Hospital (PUFH) between 2012 and 2017. Samples of cancerous tissues and adjacent normal tissues were collected from each gastric cancer patient, then the samples were frozen in liquid nitrogen immediately and kept at −80°C. Before surgical procedures, patients had submitted their written informed consent for the analysis and publication of anonymized medical data and pathological tissues for research purposes.

Tumors were retrospectively staged for the present study according to the TNM system (eighth edition) of the American Joint Committee on Cancer [29]. In addition, retrospectively collected data about each patient were obtained from the hospital database. The present study was performed according to the Declaration of Helsinki guidelines. It was authorized by the Ethics Committee on Human Research of Peking University First Hospital, and the authorization number was 2018–15.

### RNA isolation, library construction, and sequencing

Trizol reagent (Invitrogen, MA, USA) was used to extract total RNA from different kinds of cells. RNA integrity and RNA concentration were analyzed. Only RNA samples with an RNA integrity number ≥ 7.0 and a 28S:18S ratio ≥ 1.5 were used in subsequent experiments. CapitalBio Technology (Beijing, China) was applied for sequencing libraries generation. (Beijing, China). The ribosomal RNA in each sample was removed by Ribo-Zero™ Magnetic Kit (Epicentre, WI, USA). The NEBNext Ultra RNA Library Prep Kit for Illumina (NEB, MA, USA) was applied to establish the libraries for sequencing as previously described [8].

FastQC was used for determining the sequencing quality of raw data in fastq format, and NGSQC Toolkit (v2.3.3 NIPGR) was applied for low-quality data filtering. Then, clean reads of high quality were aligned to the reference genome hg38 from the University of California Santa Cruz genomics institute through Tophat2 and default parameters, and assembly of the transcripts was conducted by Cufflinks and Cuffmerge packages [30].

Based on information from the public databases, our transcripts were classified as mRNAs or lncRNAs, and mRNAs known to interact with a given lncRNA were annotated. The remaining transcripts were classified as novel lncRNAs if they were longer than 200 nt and were predicted to be non-coding.

Differential expression of RNAs between different gastric cancer cells was performed using the limma package and edgeR. Functional annotation and enrichment analyses of differentially expressed RNAs were performed using KOBAS 3.0. Target genes of differentially expressed lncRNAs were predicted based on cis- and trans-patterns in the reference genome and on sequence similarity.

### Cell culture

The human gastric cancer cell lines SGC-7901, BGC-823, HGC-27, AGS, KATO-III, MGC-803, and the normal gastric epithelial cell line GES-1 were purchased from the Cancer Institute of the Chinese Academy of Medical Science (Beijing, China). All cell lines were cultured in RPMI 1640 medium added 10% FBS. Cell culture was at 37°C with a humidified 5% CO_2_ atmosphere.

### Cell transfection

Lentiviruses encoding green fluorescent protein (GFP) cDNA and lentiviruses encoding full-length CRART16 cDNA were constructed as described [8]. Gastric cancer cells (2 × 10^5^ cells/well) were infected with different types of lentivirus in the culture medium containing 5 μg/mL polybrene (Sigma-Aldrich, MO, USA). After 24 hours, the medium was replaced with a fresh complete RPMI 1640 medium. Cultures were subjected to puromycin selection at 5.0 μg/mL for 14 days. Stably transduced SGC-7901 cells overexpressing CRART16 were named SGC-CRART16 cells, while negative control cells were denominated SGC-NC cells.

In some rescue experiments, SGC-CRART16 cells were transfected with miR-122-5p mimics (Sigma-Aldrich, MO, USA) or with short interfering RNAs (siRNAs) targeting FOS (Santa Cruz Biotechnology, New York, USA). FuGene HD Transfection Reagent (Promega, Madison, WI, USA) was used in the transient transfections.

KATO-III cells were transfected with plasmid (LV3-H1/GFP&Puro) encoding either shRNA targeting CRART16 or negative control shRNA (Shanghai GenePharma, Shanghai, China), and stable transfectants were selected using puromycin. The resulting transfectants were named KATO-shCRART16 and KATO-NC cells, respectively. The efficiency of all transfections was determined by qRT-PCR.

### Cell proliferation assay

The cell counting kit-8 (CCK-8) assay was conducted to detect cell growth rate. In brief, gastric cancer cells were seeded in 96-well plates at an approximate density of 2 × 10^4^ cells/mL. After culturing for 12, 24, 36, 48, or 60 hours, ten μL CCK-8 solution was added to each well. Subsequently, the cells were incubated at 37°C for another 2 hours. Next, the absorbance of each well was detected at 450 nm, and the cell viability was calculated as described previously [31].

### Flow cytometric analysis of apoptosis and the cell cycle

About 5.0 × 10^5^ different types of gastric cancer cells were cultured in a complete RPMI 1640 medium for 24 hours. Subsequently, cells were treated with 0.5 mg/mL bevacizumab (Avastin, Roche, Switzerland) for 48 hours. Cell apoptosis was evaluated by flow cytometry using a PE Annexin V Apoptosis Detection Kit (BD Biosciences, NJ, USA). In brief, the cells were suspended in 490 μL of annexin V binding buffer. Subsequently, 5 μL of annexin V-PE and 5 μL of propidium iodide were added to the binding buffer. All cell samples were incubated at room temperature in the dark for 5 min. Afterward, cell apoptosis was detected by a flow cytometer [31]. For cell cycle analysis, the cells were harvested and fixed in test tubes with 75% ethanol at 4°C for 12 hours. Following the centrifugation of the cells, the cells were then resuspended and stained with propidium iodide/RNase Staining Buffer (BD Biosciences, NJ, USA) as previously described [8].

### Colony formation assay

Gastric cancer cells were trypsinized, harvested, and suspended as single cells, and then they were seeded into six-well plates (500 cells/well). After 14 days, the cells were fixed with 4% paraformaldehyde for 10 min and then stained with freshly prepared 0.1% crystal violet stain for 10 min at room temperature. Colonies in each well were counted under a Canon Power Shot A640 camera on an Olympus inverted microscope with a 100× magnification. Experiments were performed in triplicate and repeated three times.

### Matrigel colony assay

As described previously [28], 14 mm microwells were precoated with 200 μL Matrigel at 11.5 mg/mL, then gastric cancer cells were plated into the wells (1.0 × 10^3^ cells/well), and then they were cultured for 14 days. The numbers of colonies (>50 μm) were determined from digital images captured under a phase-contrast light microscope and analyzed. Experiments were performed in triplicate and repeated three times.

### Endothelial cell tube formation in the presence of conditioned media

About 1.0 × 10^6^ different types of gastric cancer cells were cultured in six-well slides for 24 hours. The media of different types of cells were collected and used as media in an endothelial cell tube formation assay [32]. Briefly, the 96-well plates were pre-chilled on the ice. Subsequently, 100 μL Matrigel was carefully added to each well and allowed to set at 37°C for 30 min. The HUVECs were suspended in different kinds of conditioned media and seeded into the wells (5.0 × 10^3^ cells/100 μL/well), and then the HUVECs were cultured in the incubator at 37°C. Thirty-six hours later, the capillary-like structures were photographed. Experiments were performed in quadruplicate and repeated three times. The capillary length was measured and calculated as described previously [28].

### Spheroid sprouting assay

As previously described, a three-dimensional (3D) spheroid sprouting assay was conducted [33, 34]. In brief, HUVECs (1.0 × 10^5^ cells per well) were cultured in 96 U-well suspension plates (Corning, NY, USA). The spheroids of HUVECs were incubated in 150 μL of Endothelial Cell Growth Medium-2 (Lonza, Basel, Swiss) with 20% methylcellulose overnight, and then they were embedded into collagen gels. Subsequently, a solution of 3 mg/ml of type I collagen (BD, NJ, USA) was prepared in conditioned media harvested from different kinds of gastric cancer cells, and then the pH of the solution was neutralized by 1 M NaOH to PH 7.4. Next, the spheroids were suspended in the solution of type I collagen and incubated at 37°C for 1.5 hours. One hundred microliters of conditioned medium were added to each well following the collagen was set, and the spheroids were then cultured for another 48 hours. The sprouts of spheroids were photographed with a Canon Power Shot A640 camera as described previously [28].

### Endothelial invasion assay

The 24-well BioCoat™ Matrigel Invasion Chambers (Corning, NY, USA) were used to detect the invasive capability of endothelial cells. The lower chamber was injected with 500 μl of conditioned medium, and 1.0 × 10^4^ HUVECs suspended in 200 μL of serum-free RPMI were seeded into the upper chamber. The chamber was incubated at 37°C for 48 hours. The number of invading cells was counted in four random fields per filter. Experiments were performed in triplicate and repeated three times [28].

### Angiogenesis assay in chick chorioallantoic membranes (CAMs)

The fertilized eggs were sterilized with 75% alcohol and then cultured at 37.5°C. Seven days later, a window was opened on the shell of the eggs to expose the CAM, then was covered with a disc of filter paper (0.5 cm diameter) containing different kinds of gastric cancer cells (1.0 × 10^6^/disc) on the surface. The window was sealed with tape. Next, the eggs were incubated for another 48 hours. Afterward, the CAM was fixed in 3.7% formaldehyde, and the results were visualized under a stereoscope.

### RNA extraction and qRT-PCR

TRIzol reagent (Invitrogen, MA, USA) was applied to extract total RNA from gastric cancer cell lines and tissues following the manufacturer’s instructions and stored at −80°C. RNA concentration and purity were measured using a Nanodrop spectrophotometer. The OD260/280 ratios for all samples were between 1.8 and 2.0. Total RNA (4 μg) was reverse-transcribed into cDNA using a RevertAid RT Reverse Transcription Kit (Thermo Fisher Scientific, MA, USA). The qRT-PCR assays were conducted on an Applied Biosystems 7500 Real-Time PCR System (Applied Biosystems, CA, USA) using TransScript^®^ Green miRNA Two-Step qRT-PCR SuperMix (Transgen Biotech, Beijing, China). GAPDH and U6 small nuclear RNA were used as the internal controls. The comparative cycle threshold (2^−ΔΔCT^) method was applied to calculate relative gene expressions [28]. The primer sequences are shown in Supplementary Table 1.

### Western blotting analysis

As described previously, the cellular lysates were prepared and detected by western blotting [32]. Antibodies against (Cleaved) PARP, Pre-caspase-3, (Cleaved) caspase-3, and c-Fos were purchased from Cell Signaling Technology (MA, USA), and they were used all at a dilution of 1:500. Antibodies against vascular endothelial growth factor D (VEGFD) were obtained from Abcam (MA, USA), and they were used at a dilution of 1:500. Antibodies against GAPDH or β-Actin (CST, MA, USA) were used at a dilution of 1:1000 as a loading control.

### ELISA determinations

For the quantitative evaluation of VEGFD concentrations in conditioned media, the enzyme-linked immunosorbent assay (ELISA) was used as described previously [28]. Briefly, the VEGFD concentrations were measured using a human VEGFD ELISA kit (ab233625, Abcam, MA, USA) according to the manufacturer’s guidance. Experiments were performed in triplicate and repeated three times.

### Prediction of lncRNA targets

The miRWalk Database (http://www.mirdb.org/) was used to predict potential miRNA targets of lncRNA CRART16. The differentially expressed miRNAs from SGC-CRART16 cancer cells obtained by sequencing and those from a colon cancer cell line Caco-2-CRART16 described previously were analyzed together [8]. The overlap of differentially expressed miRNAs sets and the most probable target miRNA of CRART16 were created by the online Venn diagram plotting tool Venny 2.1.0. Subsequently, RNA22 v2 microRNA target detection and miRanda were used to identify potential base-pairing regions with the target gene.

### Construction of luciferase reporter plasmids

The 3′-UTR sequences of FOS (nt 1298-2104, Genbank accession no. NM_010234) containing one putative miR-122-5p-binding sequence (nt 1517-1536) were amplified by polymerase chain reaction (PCR). The primers used in the PCR reaction were 5′-GGAGGACCTTATCTGTGCGT-3′ (forward) and 5′-AAAGAGACACAGACCCAGGC-3′ (reverse). The PCR product was cloned into firefly luciferase reporter vector pGL3 (Promega, WI, USA) to yield pGL3-FOS-3′UTR. The full-length sequence of CRART16 was also cloned into pGL3 to yield pGL3-CRART16. The MutanBEST Kit (Takara Bio, Shiga, Japan) was used to mutate bases in the 3′-UTR of FOS and in CRART16 that bind miR-122-5p, giving rise to pGL3-Mut FOS-3′UTR and pGL3-Mut CRART16.

### Dual-luciferase reporter assay

Dual-luciferase reporter assays were conducted based on the manufacturer’s instructions for the Dual-Luciferase Reporter Assay System (Promega, WI, USA). Briefly, HEK293T cells were transfected with pGL3-FOS-3′UTR or pGL3-CRART16 plasmids, together with miR-122-5p mimics or negative control miRNA mimics (Sigma-Aldrich, MO, USA). The plasmids of pRL-TK (Promega, WI, USA) were co-transfected into the cells as a normalization control. FuGene HD Transfection Reagent (Promega, Madison, WI, USA) was used in the transient transfections. Twenty-four hours later, transfected HEK293T cells were harvested, and the activities of Firefly and Renilla luciferase were determined using a BioTek Synergy H1 Hybrid Multi-Mode Microplate Reader as described previously [8].

### Xenograft models and immunohistochemistry

Twenty-four male BALB/c nude mice (4 weeks old, 18–20 g) were maintained in a specific pathogen-free barrier facility. The mice were divided into four groups (6 mice/group), and 2.0 × 10^6^ gastric cancer cells were subcutaneously injected into the mouse’s flank. The tumors sizes were measured using a clipper every three days, and the tumor volume was calculated using the formula: Volume = 0.52× width^2^ × length [32].

As described previously, 4 μm thick serial sections were cut from the paraffin-embedded tissues of xenograft. All sections were deparaffinized and hydrated. Subsequently, the sections were incubated with primary antibodies against VEGFD, c-Fos, or CD31 at 4°C for 12 hours. IPP version 6.0 (Media Cybernetics, Silver Spring, MD) was used to assess the intensity of the stains [32].

### Statistical analysis

All Statistical analyses were conducted using SPSS 23.0 (IBM Corp., NY, USA) or Graph Pad Prism V software (GraphPad Software, CA, USA). The data in the research were displayed as mean ± standard deviation. The one-way ANOVA followed by Dunnett’s multiple comparisons was used to compare the quantitative data from three or more groups. The Student’s *t*-test was used to compare the quantitative data from two groups. Besides, the Paired Student’s *t*-test was applied to compare CRART16 expression levels in gastric cancer tissues and their corresponding normal tissues. Kaplan-Meier method was employed for OS curves plotting and analysis. *P* < 0.05 was considered statistically significant.

### Ethics approval and consent to participate

This study was conducted according to the Declaration of Helsinki guidelines and approved by the Clinical Ethics Committee of Peking University First Hospital (Approval No. 2018-15), and written informed consent was obtained from all patients. All animal experiments were approved by the Ethics Committee of Laboratory Animal Care and Use of Peking University First Hospital (Approval No. J2020-14). All animals were treated according to the standards prescribed by the “Guidelines for the welfare and use of animals in cancer research”.

### Data availability statement

The datasets analyzed during the current study are available in The Cancer Genome Atlas (http://cancergenome.nih.gov) repositories.

## Supplementary Materials

Supplementary Table 1

## References

[1] Sung H, Ferlay J, Siegel RL, Laversanne M, Soerjomataram I, Jemal A, Bray F. Global Cancer Statistics 2020: GLOBOCAN Estimates of Incidence and Mortality Worldwide for 36 Cancers in 185 Countries. CA Cancer J Clin. 2021; 71:209–49. 10.3322/caac.21660 33538338

[2] Feng RM, Zong YN, Cao SM, Xu RH. Current cancer situation in China: good or bad news from the 2018 Global Cancer Statistics? Cancer Commun (Lond). 2019; 39:22. 10.1186/s40880-019-0368-6 31030667 PMC6487510

[3] Ajani JA, Lee J, Sano T, Janjigian YY, Fan D, Song S. Gastric adenocarcinoma. Nat Rev Dis Primers. 2017; 3:17036. 10.1038/nrdp.2017.36 28569272

[4] Esteller M, Pandolfi PP. The Epitranscriptome of Noncoding RNAs in Cancer. Cancer Discov. 2017; 7:359–68. 10.1158/2159-8290.CD-16-1292 28320778 PMC5997407

[5] Wang Y, Fang Z, Hong M, Yang D, Xie W. Long-noncoding RNAs (lncRNAs) in drug metabolism and disposition, implications in cancer chemo-resistance. Acta Pharm Sin B. 2020; 10:105–12. 10.1016/j.apsb.2019.09.011 31993309 PMC6976993

[6] Li S, Zhang M, Zhang H, Hu K, Cai C, Wang J, Shi L, Ma P, Xu Y, Zheng P. Exosomal long noncoding RNA lnc-GNAQ-6:1 may serve as a diagnostic marker for gastric cancer. Clin Chim Acta. 2020; 501:252–7. 10.1016/j.cca.2019.10.047 31730812

[7] Kung JT, Colognori D, Lee JT. Long noncoding RNAs: past, present, and future. Genetics. 2013; 193:651–69. 10.1534/genetics.112.146704 23463798 PMC3583990

[8] Zhang X, Wen L, Chen S, Zhang J, Ma Y, Hu J, Yue T, Wang J, Zhu J, Bu D, Wang X. The novel long noncoding RNA CRART16 confers cetuximab resistance in colorectal cancer cells by enhancing ERBB3 expression via miR-371a-5p. Cancer Cell Int. 2020; 20:68. 10.1186/s12935-020-1155-9 32158358 PMC7057486

[9] Xu X, Gao F, Wang J, Tao L, Ye J, Ding L, Ji W, Chen X. MiR-122-5p inhibits cell migration and invasion in gastric cancer by down-regulating DUSP4. Cancer Biol Ther. 2018; 19:427–35. 10.1080/15384047.2018.1423925 29509059 PMC5915035

[10] Chen Q, Ge X, Zhang Y, Xia H, Yuan D, Tang Q, Chen L, Pang X, Leng W, Bi F. Plasma miR-122 and miR-192 as potential novel biomarkers for the early detection of distant metastasis of gastric cancer. Oncol Rep. 2014; 31:1863–70. 10.3892/or.2014.3004 24481716

[11] Jiao Y, Zhang L, Li J, He Y, Zhang X, Li J. Exosomal miR-122-5p inhibits tumorigenicity of gastric cancer by downregulating *GIT1*. Int J Biol Markers. 2021; 36:36–46. 10.1177/1724600821990677 33752480

[12] Hong H, Jiang L, Lin Y, He C, Zhu G, Du Q, Wang X, She F, Chen Y. TNF-alpha promotes lymphangiogenesis and lymphatic metastasis of gallbladder cancer through the ERK1/2/AP-1/VEGF-D pathway. BMC Cancer. 2016; 16:240. 10.1186/s12885-016-2259-4 26992854 PMC4799527

[13] Ming J, Zhang Q, Qiu X, Wang E. Interleukin 7/interleukin 7 receptor induce c-Fos/c-Jun-dependent vascular endothelial growth factor-D up-regulation: a mechanism of lymphangiogenesis in lung cancer. Eur J Cancer. 2009; 45:866–73. 10.1016/j.ejca.2008.12.006 19136250

[14] Weekes D, Kashima TG, Zandueta C, Perurena N, Thomas DP, Sunters A, Vuillier C, Bozec A, El-Emir E, Miletich I, Patiño-Garcia A, Lecanda F, Grigoriadis AE. Regulation of osteosarcoma cell lung metastasis by the c-Fos/AP-1 target FGFR1. Oncogene. 2016; 35:2852–61. 10.1038/onc.2015.344 26387545 PMC4688957

[15] Muhammad N, Bhattacharya S, Steele R, Phillips N, Ray RB. Involvement of c-Fos in the Promotion of Cancer Stem-like Cell Properties in Head and Neck Squamous Cell Carcinoma. Clin Cancer Res. 2017; 23:3120–8. 10.1158/1078-0432.CCR-16-2811 27965308 PMC5468504

[16] Atsaves V, Leventaki V, Rassidakis GZ, Claret FX. AP-1 Transcription Factors as Regulators of Immune Responses in Cancer. Cancers (Basel). 2019; 11:E1037. 10.3390/cancers11071037 31340499 PMC6678392

[17] Ding Y, Hao K, Li Z, Ma R, Zhou Y, Zhou Z, Wei M, Liao Y, Dai Y, Yang Y, Zhang X, Zhao L. c-Fos separation from Lamin A/C by GDF15 promotes colon cancer invasion and metastasis in inflammatory microenvironment. J Cell Physiol. 2020; 235:4407–21. 10.1002/jcp.29317 31613004

[18] Huang Q, Lan F, Wang X, Yu Y, Ouyang X, Zheng F, Han J, Lin Y, Xie Y, Xie F, Liu W, Yang X, Wang H, et al. IL-1β-induced activation of p38 promotes metastasis in gastric adenocarcinoma via upregulation of AP-1/c-fos, MMP2 and MMP9. Mol Cancer. 2014; 13:18. 10.1186/1476-4598-13-18 24479681 PMC3937117

[19] Orlandini M, Marconcini L, Ferruzzi R, Oliviero S. Identification of a c-fos-induced gene that is related to the platelet-derived growth factor/vascular endothelial growth factor family. Proc Natl Acad Sci U S A. 1996; 93:11675–80. 10.1073/pnas.93.21.11675 8876195 PMC38117

[20] Pan T, Jin Z, Yu Z, Wu X, Chang X, Fan Z, Li F, Wang X, Li Z, Zhou Q, Li J, Liu B, Su L. Cathepsin L promotes angiogenesis by regulating the CDP/Cux/VEGF-D pathway in human gastric cancer. Gastric Cancer. 2020; 23:974–87. 10.1007/s10120-020-01080-6 32388635 PMC7567730

[21] Schimanski CC, Schlaegel F, Jordan M, Moehler M, Sgourakis G, Drescher DG, Galle PR, Lang H, Gockel I. VEGF-D correlates with metastatic disease in gastric cancer patients undergoing surgery. World J Surg. 2011; 35:1010–6. 10.1007/s00268-011-1041-7 21387130

[22] Niu G, Chen X. Vascular endothelial growth factor as an anti-angiogenic target for cancer therapy. Curr Drug Targets. 2010; 11:1000–17. 10.2174/138945010791591395 20426765 PMC3617502

[23] Ferrara N, Hillan KJ, Gerber HP, Novotny W. Discovery and development of bevacizumab, an anti-VEGF antibody for treating cancer. Nat Rev Drug Discov. 2004; 3:391–400. 10.1038/nrd1381 15136787

[24] Ninomiya S, Inomata M, Tajima M, Ali AT, Ueda Y, Shiraishi N, Kitano S. Effect of bevacizumab, a humanized monoclonal antibody to vascular endothelial growth factor, on peritoneal metastasis of MNK-45P human gastric cancer in mice. J Surg Res. 2009; 154:196–202. 10.1016/j.jss.2008.08.017 19329124

[25] Shah MA, Jhawer M, Ilson DH, Lefkowitz RA, Robinson E, Capanu M, Kelsen DP. Phase II study of modified docetaxel, cisplatin, and fluorouracil with bevacizumab in patients with metastatic gastroesophageal adenocarcinoma. J Clin Oncol. 2011; 29:868–74. 10.1200/JCO.2010.32.0770 21189380 PMC3646322

[26] Lieu CH, Tran H, Jiang ZQ, Mao M, Overman MJ, Lin E, Eng C, Morris J, Ellis L, Heymach JV, Kopetz S. The association of alternate VEGF ligands with resistance to anti-VEGF therapy in metastatic colorectal cancer. PLoS One. 2013; 8:e77117. 10.1371/journal.pone.0077117 24143206 PMC3797099

[27] Patil I. ‘ggplot2’ Based Plots with Statistical Details (R package ggstatsplot version 0.0.1). 2018.

[28] Zhang J, Zhang J, Pang X, Chen Z, Zhang Z, Lei L, Xu H, Wen L, Zhu J, Jiang Y, Cui Y, Chen G, Wang X. MiR-205-5p suppresses angiogenesis in gastric cancer by downregulating the expression of VEGFA and FGF1. Exp Cell Res. 2021; 404:112579. 10.1016/j.yexcr.2021.112579 33957117

[29] In H, Solsky I, Palis B, Langdon-Embry M, Ajani J, Sano T. Validation of the 8^th^ Edition of the AJCC TNM Staging System for Gastric Cancer using the National Cancer Database. Ann Surg Oncol. 2017; 24:3683–91. 10.1245/s10434-017-6078-x 28895113

[30] Trapnell C, Roberts A, Goff L, Pertea G, Kim D, Kelley DR, Pimentel H, Salzberg SL, Rinn JL, Pachter L. Differential gene and transcript expression analysis of RNA-seq experiments with TopHat and Cufflinks. Nat Protoc. 2012; 7:562–78. 10.1038/nprot.2012.016 22383036 PMC3334321

[31] Zhang JL, Liu XZ, Wang PY, Chen GW, Jiang Y, Qiao SK, Zhu J, Wang X, Pan YS, Liu YC. Targeting HCCR expression resensitizes gastric cancer cells to chemotherapy via down-regulating the activation of STAT3. Sci Rep. 2016; 6:24196. 10.1038/srep24196 27052330 PMC4823702

[32] Zhang JL, Chen GW, Liu YC, Wang PY, Wang X, Wan YL, Zhu J, Gao HQ, Yin J, Wang W, Tian ML. Secreted protein acidic and rich in cysteine (SPARC) suppresses angiogenesis by down-regulating the expression of VEGF and MMP-7 in gastric cancer. PLoS One. 2012; 7:e44618. 10.1371/journal.pone.0044618 22957090 PMC3434168

[33] Maracle CX, Kucharzewska P, Helder B, van der Horst C, Correa de Sampaio P, Noort AR, van Zoest K, Griffioen AW, Olsson H, Tas SW. Targeting non-canonical nuclear factor-κB signalling attenuates neovascularization in a novel 3D model of rheumatoid arthritis synovial angiogenesis. Rheumatology (Oxford). 2017; 56:294–302. 10.1093/rheumatology/kew393 27864565

[34] Wu XG, Zhou CF, Zhang YM, Yan RM, Wei WF, Chen XJ, Yi HY, Liang LJ, Fan LS, Liang L, Wu S, Wang W. Cancer-derived exosomal miR-221-3p promotes angiogenesis by targeting THBS2 in cervical squamous cell carcinoma. Angiogenesis. 2019; 22:397–410. 10.1007/s10456-019-09665-1 30993566

