# Supplementary material for: LncRNA CRART16/miR-122-5p/FOS axis promotes angiogenesis of gastric cancer by upregulating VEGFD expression
Source: Aging (Albany NY). 2022 May 10;14(9):4137–57. doi: 10.18632/aging.204078 (PMC9134963; doi:10.18632/aging.204078)
Supplement: Supplementary Table 1 [file aging-14-204078-s001.pdf]

SUPPLEMENTARY TABLE

Supplementary Table 1. Primers used for quantitative real time PCR.

Primer	Sequence
CRART16 Forward	5′-TGATAGTGAGGCCTCCTGCAA-3′
CRART16 Reverse	5′-CTGGAGTTCTGCAGGTTCCTTT-3′
miR-122-5p Forward	5′-GCTGTGGAGTGTGACAATGGTGTTTG-3′
U6 Forward	5′-GCAAGGATGACACGCAAATTC-3′
FOS Forward	5′-CAAGCGGAGACAGACCAACT-3′
FOS Reverse	5′-TGAGCCGCTAGGATGAACTC-3′
VEGFD Forward	5′-ATCTGTATGAACACCAGCACCTC-3′
VEGFD Reverse	5′-TGGCAACTTTAACAGGCACTAAT-3′
GAPDH Forward	5′-ATGGGGAAGGTGAAGGTCGG-3′
GAPDH Reverse	5′-GACGGTGCCATGGAATTTGC-3′
